# BAC-Pool Sequencing and Analysis of Large Segments of A12 and D12 Homoeologous Chromosomes in Upland Cotton

**DOI:** 10.1371/journal.pone.0076757

**Published:** 2013-10-08

**Authors:** Ramesh Buyyarapu, Ramesh V. Kantety, John Z. Yu, Zhanyou Xu, Russell J. Kohel, Richard G. Percy, Simone Macmil, Graham B. Wiley, Bruce A. Roe, Govind C. Sharma

**Affiliations:** 1 Center for Molecular Biology, Department of Biological and Environmental Sciences, Alabama Agricultural & Mechanical University, Normal, Alabama, United States of America; 2 United States Department of Agriculture, Agricultural Research Service, Southern Plains Agricultural Research Center, Crop Germplasm Research Unit, College Station, Texas, United States of America; 3 Advanced Center for Genome Technology, University of Oklahoma, Norman, Oklahoma, United States of America; 4 Gene Structure and Function Laboratory, University of Otago, Dunedin, New Zealand; 5 Arthritis & Immunology Department, Oklahoma Medical Research Foundation, Oklahoma City, Oklahoma, United States of America; New Jersey Institute of Technology, United States of America

## Abstract

Although new and emerging next-generation sequencing (NGS) technologies have reduced sequencing costs significantly, much work remains to implement them for *de novo* sequencing of complex and highly repetitive genomes such as the tetraploid genome of Upland cotton (*Gossypium hirsutum* L.). Herein we report the results from implementing a novel, hybrid Sanger/454-based BAC-pool sequencing strategy using minimum tiling path (MTP) BACs from Ctg-3301 and Ctg-465, two large genomic segments in A12 and D12 homoeologous chromosomes (Ctg). To enable generation of longer contig sequences in assembly, we implemented a hybrid assembly method to process ~35x data from 454 technology and 2.8-3x data from Sanger method. Hybrid assemblies offered higher sequence coverage and better sequence assemblies. Homology studies revealed the presence of retrotransposon regions like *Copia* and *Gypsy* elements in these contigs and also helped in identifying new genomic SSRs. Unigenes were anchored to the sequences in Ctg-3301 and Ctg-465 to support the physical map. Gene density, gene structure and protein sequence information derived from protein prediction programs were used to obtain the functional annotation of these genes. Comparative analysis of both contigs with *Arabidopsis* genome exhibited synteny and microcollinearity with a conserved gene order in both genomes. This study provides insight about use of MTP-based BAC-pool sequencing approach for sequencing complex polyploid genomes with limited constraints in generating better sequence assemblies to build reference scaffold sequences. Combining the utilities of MTP-based BAC-pool sequencing with current longer and short read NGS technologies in multiplexed format would provide a new direction to cost-effectively and precisely sequence complex plant genomes.

## Introduction

Cotton is one of the most important fiber and oil seed crops and it contributes ~ $500 billion/yr. to world’s economy [1]. The ‘*Gossypium*’ genus consists of nearly 50 different cotton species including five allotetraploids (A_t_D_t_) and other diploids. Cotton fiber has been studied widely to understand the cell elongation and cellulose synthesis. The need to understand the genome organization, complexity and evolution of cotton has provided an impetus to sequencing efforts, which have gained momentum in recent years. Decoding cotton genomes continues to be a quest for understanding of the functional and agronomic significance of ploidy and genome size variation within the *Gossypium* genus [1]. However, choosing a cost-effective sequencing strategy that can deliver informative whole genome sequence is of major concern for many polyploid species.

The most extensively cultivated cotton species, *Gossypium hirsutum* L., is an allotetraploid and accounts for 90% of world cotton production. *G. hirsutum* belongs to AD_1_ genome and comprises an approximate haploid genome (1C) size of ~2,425 Mb [2]. Diverse molecular markers have been used to develop an integrated, high density genetic and physical map using *G. hirsutum* as one of the parents in several mapping populations [3-8]. The International Cotton Genome Initiative (ICGI) had proposed to develop a physical map of Upland cotton using TM-1 (*G. hirsutum*) as the genetic standard [9]. Availability of physical map anchored with Bacterial Artificial Chromosome (BAC) information would improve the ability to decipher the genome sequence more precisely and to assign sequences to individual sub-genomes in the tetraploid cotton genomes.

Genome sequencing of crop plants is very important for crop improvement through efficient breeding and gene discovery [10]. Eukaryotic plant genomes exhibit extraordinary genome size variation due to the proliferation of transposons and retroposons in addition to polyploidy. Until recently, whole genome shotgun sequencing (WGS) by Sanger sequencing method was the main approach for sequencing the large genomes [11]. The advent of next-generation sequencing (NGS) technologies changed the dynamics and ramped up the pace of genomic research in human, plants, animals and microorganisms because of their rapid, inexpensive and highly accurate sequencing capabilities. Existing NGS technologies, including pyrosequencing (Roche 454 GS FLX), sequencing-by-synthesis (Illumina HiSeq2000), sequencing-by-ligation (Life Technologies SOLiD 5500xl), single molecule sequencing (Pacific Biosciences RS, Helicos Biosciences HeliScope), and others provide an opportunity to sequence large and complex genomes in a rapid and cost-effective manner. For *de novo* sequencing of complex polyploid genomes such as cotton, the read length in the sequence data plays a pivotal role in the data assembly and analysis. Though the short read technologies (e.g. HiSeq 2000 and SOLiD 5500xl) provide the lowest cost/base, their application for *de novo* sequencing and assembly of large genomes is limited.

Roche 454 pyrosequencing technology (454 GS 20) was first introduced in 2005 [12] with an average read length of ~100 bases, but the recent version of this instrument (454/Roche Genome Sequencer FLX Plus with Titanium XL+ chemistry) is capable of generating more than one million reads with improved N50 read length of 700-800 bases per 24-hour instrument run. The 454 sequencing has been employed successfully in wide array of applications including *de novo* sequencing and re-sequencing applications for whole genome sequencing of microbial, fungal, viral genomes; transcriptome sequencing, serial analysis of gene expression (SAGE); cDNA/EST sequencing; metagenomics applications; methylation analysis [13-18]. Despite the high throughput capabilities of existing NGS platforms, sequencing accuracy is still of concern to the research community. Dependency on any one approach to genome-wide sequencing of large eukaryotic genomes presents several challenges and limitations.

To expedite and deduce high quality draft assemblies in an effective manner, we proposed a novel BAC-pool based strategy to sequence two large contig regions in cotton using a Sanger-454 hybrid approach [19]. From the two well-characterized homoeologous chromosomes in cotton [9], two large contig (Ctg) regions: Ctg-3301 spanning ~767Kb from chromosome A12 (chromosome 12) and Ctg-465 spanning ~1,540Kb from chromosome D12 (chromosome 26) were selected to demonstrate our sequencing strategy. The major objectives of this study include (i) develop a cost-effective yet efficient strategy to sequence the cotton genomes using minimal tiling path (MTP) of BAC libraries, (ii) evaluate the efficiency of the approach for generating high quality *de novo* sequence assemblies, (iii) analyse the sequence assembly for the presence of genetic markers, genome specific repeats, coding capacity, and (iv) comparative analysis of the contigs with *Arabidopsis* genome for gene discovery and synteny. The BAC clones were selected based on a MTP approach to improve the data quality while reducing the redundancy. This study would not only help evaluate the efficiency of sequencing strategy, but it would also provide a future direction for sequencing complex polyploid genomes.

## Materials and Methods

### Contig selection

Large insert BAC and plant transformation competent binary large insert plasmid clone (BIBAC) libraries were constructed for TM-1 (*G. hirsutum*) using partial digestions with restriction enzymes *Bam*HI and *Hind*III at USDA-ARS facility, College Station, TX [20]. The *Bam*HI library was cloned into a BIBAC vector (pCLD04541) and transformed into *E. coli* strain DH10B. Based on the BAC finger printing data, overlapping BAC clones in a MTP were selected to form small pools covering 0.5-1Mb contig regions. Selecting similar contiguous overlapping BAC clones in pools extended them to longer contig regions. Based on MTP, seven and twelve BACs were selected for sequencing from Ctg-3301 and Ctg-465. Ctg-3301 contained Pool-4 and Pool-5 with 4 and 3 BAC clones, respectively; while Ctg-465 comprised of Pool-1, Pool-2 and Pool-3 each with 4 BAC clones.

### Sequencing

TM-1 inserts from the BAC clones in a pool were isolated individually using a modified cleared lysate protocol described earlier [21]. After pooling, the BAC DNA was sheared, concentrated, end-repaired, adapters were ligated, and after another round of end-repair, the blunt ended DNA was ligation to microbeads in 1:1 ratio as described earlier [22]. After emulsion-based amplified fragments, each BAC-pool was loaded separately onto a quarter of PicoTiter plates for 454/Roche GS-FLX pyrosequencing. Similarly isolated DNA from seven Ctg-3301 BACs and from ten Ctg-465 BACs were sequenced individually to 2.8-3.0 fold coverage using ABI 3730xl capillary sequencers by paired-end method [23]. Sanger and 454 sequencing were performed at Advanced Center for Genome Technology (ACGT), Norman, OK.

### Sequence Assembly

The sequence data from 454 was assembled using the 454/Roche Newbler assembler [12] for raw untrimmed reads, reads trimmed to 84 flow cycles and reads trimmed to 63 flow cycles to improve the accuracy of homopolymer repeats (unpublished observation). These three independent assemblies then were merged using the Phrap with high stringency assembly conditions of double default values of min_match=30 and min_score=55 for respective BAC-pool “triple assembly and this triple assembly improved assembly of homopolymer repeat regions. ABI-Sanger raw data also was assembled using Phrap [24] to generate sequence contigs for each BAC clone. 454 raw reads in a BAC-pool were assigned with a minimum putative ‘Phred’ quality value of 16 to use them along with the ABI reads from the respective BAC clones to generate Sanger-454 hybrid pool assemblies using Phrap. All the assemblies were generated at the ACGT facility, Norman, OK and are available at GenBank with accession numbers as presented in Table 1.

**Table 1:** Summary of 454, ABI-Sanger and hybrid assemblies for Ctg-465 and Ctg-3301 in cotton.

			ABI-Sanger Assembly			454 Assembly			Sanger-454 Hybrid Pool Assembly		
Ctg/BAC-Pool	BAC Clone	Estimated TM-1 insert size in Kb	GenBank ID	No. of Contigs	Assembly Length	GenBank ID	No. of Contigs	Assembly Length	GenBank ID	No. of Contigs	Assembly Length
Pool-4	CBV101N18	125	AC245509	16	109,312	-	158	208,437	SRR346495	87	203,770
	CBV103A21	130	AC245640	13	126,266						
	CBV056O13	85	AC245649	18	164,636						
	CBV102M21	90	AC245508	23	133,998						
Pool-5	CBV103L18	120	AC245652	46	114,868	-	173	130,029	SRR346497	100	189,133
	CBV123K05	150	-	30	31,469						
	CBV198M14	90	AC245641	16	112,554						
**Ctg-3301 in Chromosome A12**		**~513**		**162**	**793,103**		**331**	**338,466**		**187**	**392,903**
Pool-1	CBV133F04	97	AC245642	26	221,760	-	55	227,629	SRR346488	54	241,782
	CBV064M05	100	AC245654	18	148,473						
	CBV165G03	100	AC245655	16	174,508						
	CBV103I14	105	AC245648	29	150,308						
Pool-2	CBV004P17	110	AC245643	15	138,762	-	139	252,198	SRR346491	70	275,267
	CBV155K01	160	AC245653	0	0						
	CBV015C19	110	AC245645	12	131,490						
	CBV188K21	90	AC245644	15	139,937						
Pool-3	CBV006G16	120	AC245651	34	108,719	-	307	194,195	SRR346493	79	218,525
	CBV031N01	110	AC245647	0	0						
	CBV076H08	160	AC245650	23	135,599						
	CBV176F03	120	AC245646	30	173,886						
**Ctg-465 in Chromosome D12**		**~898**		**218**	**1,523,442**		**501**	**674,022**		**203**	**735,574**

### Evaluation of de novo sequence assemblies

BAC-end sequences (BES) for all the BAC clones in this study were generated and provided by USDA-ARS, College Station, TX. Homology searches for these sequences were performed across 454, Sanger and hybrid assemblies using BLASTN program (ftp://ftp.ncbi.nih.gov/blast/executables/LATEST/) locally [25]. Sequence homology with BES in these regions aided in estimating the efficiency of each assembly process and in predicting the orientation of BACs in the pools. The anchoring marker information for chromosome A12 and D12 in cotton [9] were used for sequence homology across the assemblies using local BLAST program to identify potential molecular markers (e-value < e^-20^) specific to the sequenced regions.

### Sequence analysis

Detection of repeat elements was carried out using the ‘Censor’ program [26] at http://www.girinst.org/censor/index.php using *Arabidopsis* and dicots as reference repeat database. EST, CN, GSS sequences were obtained from GenBank for 4 important *Gossypium* species [*G. hirsutum* (265,815 ESTs, 49906 GSS, 3,897 CN); *G. barbadense* (1,023 ESTs, 57 GSS, 642 CN); *G. arboreum* (39,232 ESTs, 659 GSS, 110 CN) and *G. raimondii* (63,577 ESTs, 3,363 GSS, 464 CN)] and were used as a query against 454, ABI, and hybrid pool assemblies to derive the homologous sequence information using BLASTN program. SSR marker information was obtained from CMD (www.cottonmarker.org) for *in silico* PCR; and ‘Sputnik’ program was used for finding SSR motifs in the assembled contigs. FGENESH program was used for *ab initio* gene prediction using *Arabidopsis* as reference. Gene annotation and putative gene function was derived using the BLAST2GO application [27] at www.blast2go.org. Comparative genomic study was conducted using the GenomeVISTA tools [28] at http://genome.lbl.gov/vista/index.shtml.

## Results

### BAC-pool selection in A12 and D12 contigs

From the integrated physical maps in cotton [9], Ctg-3301 on chromosome **A12** and Ctg-465 on homoeologous chromosome **D12** were selected randomly to demonstrate our strategy. Multiple contiguous BAC clones in a MTP that covered a larger region were composed into small BAC-pools with 3-4 BACs/pool in each contig. Based on MTP, seven and twelve BACs were selected for sequencing from Ctg-3301 (A12) and Ctg-465 (D12) respectively. Ctg-3301 was composed with Pool-4 and Pool-5 with 4 and 3 BAC clones respectively; while Ctg-465 comprised of Pool-1, Pool-2 and Pool-3 each with 4 BAC clones.

### DNA sequencing

**Figure 1:**
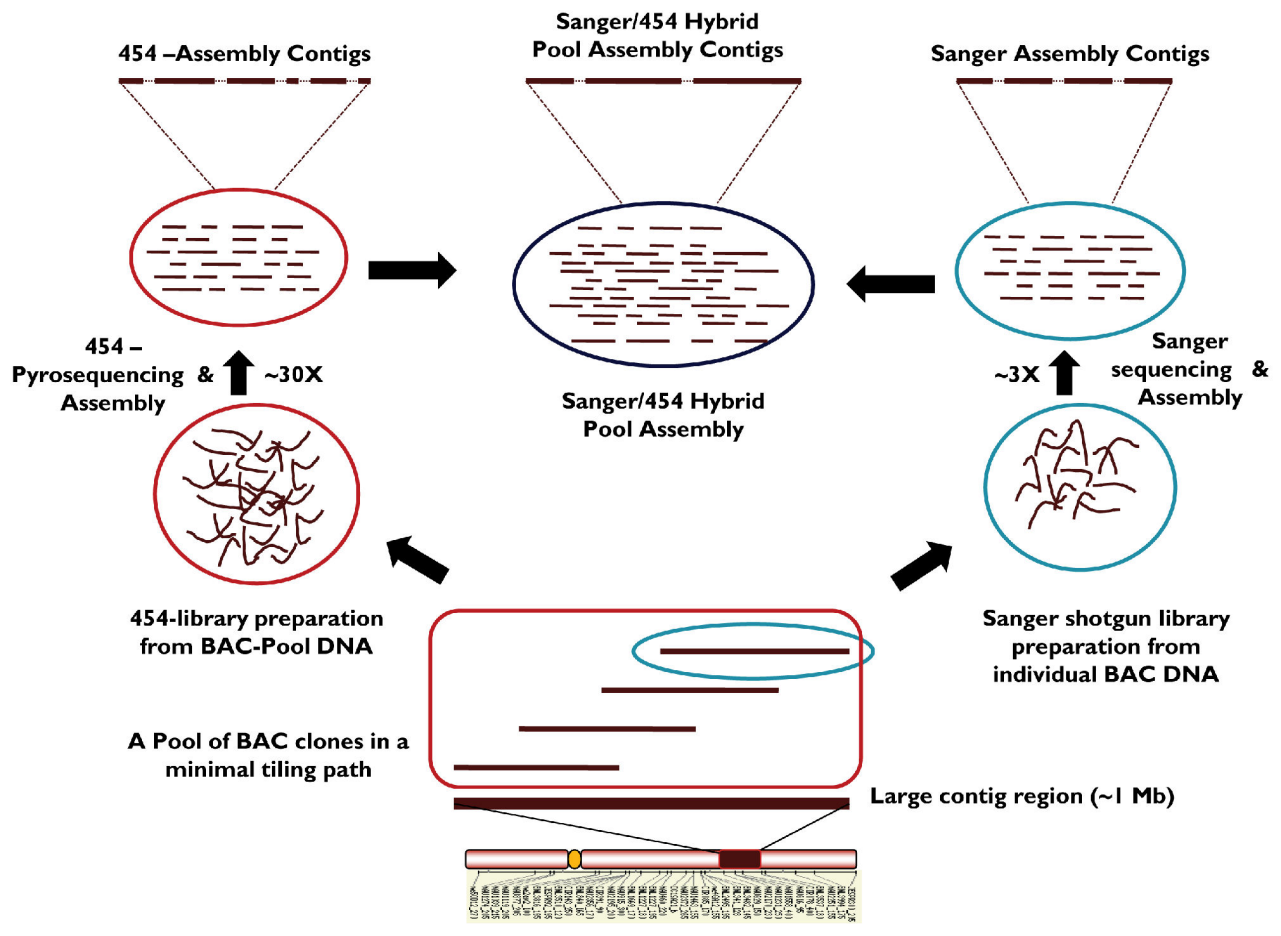
A schematic diagram representing MTP based BAC-pool sequencing for complex genomes. 454 and Sanger hybrid sequencing approach was used to derive high quality sequence assemblies in allotetraploid cotton.

To demonstrate the MTP-based BAC-pool sequencing strategy (Figure 1), Ctg-3301 and Ctg-465 were put together into small BAC pools each with 3-4 BACs/Pool and were sequenced using 454 GS FLX instrument. In 1.25 times of a full PicoTiterPlate^TM^ run, 454/Roche GS-FLX sequencer generated 49,230,429 bases of raw sequence data from 246,022 reads and with an average read length of >200 bp. The raw sequence data (~17.7 Mb) was generated from 96,224 reads providing ‘~34.5X’ coverage for Ctg-3301(~513.5 Kb) and ‘~35X’ coverage from ~31.5 Mb of raw data in 149,798 sequences from Ctg-465; while Ctg-465(~898.2Kb) region was sequenced up to ‘~35X’ coverage from ~31.5Mb of raw data in 149,798 sequences. The raw data was submitted to NCBI Short Read Archive (SRA) database (GenBank SRA Accessions: SRR346488, SRR346491, SRR346493, SRR346495, SRR346497).

Similarly for comparison and improved assembly purposes, a total of 17 BAC clones were sequenced at a lower coverage (~3X) using Sanger’s sequencing method to generate longer raw sequences with an average read length of >800 bases. CBV155K01 and CBV031N01 from Ctg-465 and CBV123K05 from Ctg-3301were not sequenced as these were hidden BAC clones (completely covered by other BACs in the contig based on BAC finger printing data) in their respective pools. CBV123K05 from Ctg-3301was also sequenced at very low coverage for a similar reason. Both 454 and Sanger sequencing were conducted at Advanced Center for Genome Technology (ACGT), Norman, OK. The BAC-pool, individual BAC clones in each pool, and sequence assembly statistics were summarized in Table 1 (GenBank Accessions: AC245508-AC245509; AC245640-AC245655).

### Sequence Assembly

Ctg-3301 was assembled into 331 contigs ranging from 100bp to ~46.8Kb and comprising a total of 338,466 bases of assembled data. Ctg-465 was assembled into 674,022 bases of assembled data in 501 contigs ranging from ~120 bases to ~75Kb in size. In these assemblies, a larger percent (>50%) of the contigs were <200bp while only <1% of the contigs were >30Kb. The GC% in these assembled pools ranged from 34-36.3% and the cumulative assembly covered ~66% and ~75% of estimated sizes of Ctg-3301 and Ctg-465 respectively. Sanger data also were assembled using Phrap [24] to generate sequence contigs for each BAC clone. When paired-end reads were included in the assembly, longer and fewer contigs were generated for each BAC. A total of 162 contigs were formed with a cumulative assembly size of 793,103 bases for Ctg-3301 from 7 individual BAC assemblies, while 218 contigs were generated with the cumulative assembly size of 1,523,442 bases from 10 individual BAC clone assemblies in Ctg-465.

As both 454 and Sanger data were derived from the same BACs, we had evaluated the hybrid assembly approach for better assembly generation for individual BAC-pools and were also tested with random permutation. Random permutation of reads at 25%, 50%, 75% and 100% in a BAC pool dataset had minimal effect on the assembly size. The number of contigs was approximately similar at 75% and 100% of reads. N50 read lengths were greatly improved from 50% of reads to 100% of reads. Ctg-3301 was assembled into 187 contigs with an increased cumulative assembly size (~393Kb) compared to 454-assembly. For Ctg-465, the cumulative assembly size was also increased to ~735Kb with a reduced number (203) of contigs. Sequence assembly statistics and a comparative diagram of the cumulative assembly length of Ctg-3301 and Ctg-465 in both 454 and hybrid (454 reads + Sanger reads) assemblies with the estimated contig size were summarized in file S1. The contigs from BAC-pools as well as individual BAC clones were submitted to NCBI Genome Survey Sequence (GSS) database.

### Evaluation of sequence assemblies

The performance of each sequencing and assembly approach was evaluated individually and jointly using BLAST homology search for the BAC-end sequences (BES) and anchored markers to the integrated physical maps of chromosomes A12 and D12 in these assemblies. For Ctg-3301, 90 and 26% of homology with forward and reverse BES sequences was observed in 454-assemblies, and 89 and 13% for Sanger assemblies. Similarly, for Ctg-465, 83 and 43% of homology with forward and reverse BES was observed in 454-assemblies, while and 73 and 31% Sanger assemblies. Lower sequence coverage BES in Sanger compared to 454-assemblies was due to the lower coverage (~3X) of sequencing of BAC clones. For both Ctg-3301 and Ctg-465 in hybrid assemblies, ± 1% change in homology compared to 454-pool assemblies was observed. In both 454 and hybrid assemblies, we also observed coverage of the BES in pools other than their parent pools as these pools overlapped with each other. Though there was minimal improvement in homology with BAC-end sequences, great improvement was observed in average contig size and reduced number of contigs (fewer gaps) with hybrid assemblies compared to 454 data derived assembly. The overlapping patterns between the BAC-pools in Ctg-3301 and Ctg-465 were diagrammatically represented using ‘Circos’ [29] in file S2.

### Sequence analysis

#### GC content and repeat elements

Contigs derived from the hybrid assembly (HA) of each BAC-pool were used for all further sequence analyses. The assembled contigs were used to calculate the GC content and was ranged between 33.3-35.8% across the BAC-pools in both Ctg-3301 and Ctg-465. Censor [26], a tool to screen and annotate repeat elements, was used to identify and annotate the repeat elements, based on the *Arabidopsis* repeat database as reference, in Ctg-3301 and Ctg-465 (File S3). LTR retrotransposon elements such as *Copia* and *Gypsy* elements were the most abundant type of repeats present in both Ctg-3301 and Ctg-465. The HA contigs were masked for these repeat regions and were used for further analyses such as finding simple sequence repeats (SSR), homology search, marker analysis, gene prediction.

#### Species specific homologies and SSR identification

HA contigs from Ctg-3301 and Ctg-465 were searched for homology with the core nucleotide (CN), expressed sequence tags (EST), genome survey sequences (GSS) of four *Gossypium* species vs. *G. hirsutum* (AD_1_), *G. barbadense* (AD_2_), *G. arboreum* (A) and *G. raimondii* (D) in GenBank. Most of the significant hits were derived from the *G. hirsutum* species as the BAC clones were derived from TM-1, a *G. hirsutum* genotype, and also *G. hirsutum* sequences were the major data resource available among the four species in GenBank (as of September 2011). High sequence similarity was observed with GSS database compared to EST and CN databases across all the pools (File S4). HA contigs also were searched for SSR motifs as they would contribute high value to generate new marker resources for these chromosomal regions. SSR motifs were identified using ‘Sputnik’ program [30] and interestingly more penta-nucleotide repeat motifs were abundant compared to di, tri, tetra repeat motifs. Repeat type, SSR motif and related information in each BAC-pool HA contigs were summarized in File S4.

#### SSR Marker and Unigene Analysis

Cotton Microsatellite Database (CMD) hosts the marker information in cotton from multiple resources including academic and industrial sectors [31]. Using the SSR marker (primer) information at CMD, *in silico* PCR was conducted to identify potential targets for amplification in the HA contigs. There were six SSR marker putative targets in Ctg-3301 and four SSR marker putative targets in Ctg-465 and this information would help localization of those marker fragment lengths to A12 and D12 chromosomes (File S5).

Comparison of chromosome D12 anchored unigene/marker sequences [9] with the HA contigs resulted in having significant homology with 31 fiber unigenes and 15 non-fiber unigenes in Pool-1, 3 fiber genes in Pool-2 and 1 fiber gene to Pool-3 in Ctg-465. Similarly, 13 non-fiber unigenes and 6 fiber unigenes in Pool-4, and 4 non-fiber related and 7 fiber related unigenes in Pool-5 in Ctg-3301 also found significant homology with chromosome A12 anchored markers.

#### Gene prediction and annotation

Availability of gene information from both Ctg-3301 and Ctg-465 helps in calculating gene density, gene structure including intron-exon information, and putative gene function. FGENESH, an *ab initio* gene finding program [32], was used to predict the gene information using *Arabidopsis* and other dicots as reference gene information. The number of protein coding regions was 48, 42, 47, 33, and 44 in BAC-pool 1, 2, 3, 4 and 5 respectively. There were a total of 71 non-redundant (nr) protein predictions detected in Ctg-3301 while 109 ‘nr’ protein predictions were detected in Ctg-465. These predicted protein sequences were searched with ‘nr’ protein database at GenBank to derive the gene function using ‘BLAST2GO’ tools [27]. Forty eight predicted proteins were annotated with gene name and protein function individually in Ctg-3301 and Ctg-465. The un-annotated predicted proteins in both Ctg-3301 and Ctg-465 were considered as hypothetical proteins with unknown function. Predicted proteins, their sequence, and annotation information in each BAC-pool was tabulated in File S6.

#### Comparison with *Arabidopsis* Genome

Cotton is one of the closest relatives of *Arabidopsis* outside the order *Brassicales* [33]. HA contigs from all five BAC pools were compared with *Arabidopsis* genome using VISTA [28] tools at http://genome.lbl.gov/cgi-bin/GenomeVista. This revealed homologous regions of Ctg-3301 and Ctg-465 in chromosome sequences of *Arabidopsis* genome and also provided the microcollinearity in the order of the genes in syntenic regions. Comparison of the sequences from Ctg-3301 and Ctg-465 with *Arabidopsis* genome helped in identifying syntenic regions and in understanding the gene structure and gene evolution.

## Discussion

Improvements in crop productivity require use of elite and wild germplasm resources along with the adoption of new breeding technologies. Genome sequencing of model plants and crop species provide opportunities to understand the genome organization, evolution, molecular characterization of genes underlying the important biological and agronomic traits. These efforts would also expedite generation of new molecular resources for improved breeding practices for crop improvement. Traditionally, whole genome sequencing efforts were assisted by shotgun sequencing of individual BAC-clones and assemblies were aligned to the chromosomes based on a BAC-based physical map [34]. Traditional BAC-by-BAC sequencing approach to sequence small plant genomes such as *Arabidopsis* (~145Mb) was economically viable using Sanger method and provide the benefit of longer and paired-end reads that serve to generate better assemblies. However, traditional approaches are very expensive, time-consuming compared to WGS methods. Though WGS methods are rapid means to generate sequencing data, significant challenges will be posed in assembly of regions containing multigene families and repetitive elements.

Next-generation sequencing technologies have emerged in recent years and are promising cost-effective and high quality sequence data in less turnaround time while simplifying multiple applications such as genome sequencing, gene discovery, genotyping, clinical research, diagnostics and others. Multiple angiosperm genome sequencing and re-sequencing projects have been initiated or currently in progress or completed in past few years. Recently, a draft genome of *G. raimondii*, a diploid D-genome progenitor of tetraploid cotton was also generated by multiple NGS methods [35,36]. Small to medium size genomes can be readily *de novo* sequenced with the current NGS platforms using whole genome shotgun sequencing methods. Unlike diploid plant species, tetraploid cotton genome is highly complex, highly repetitive with minimal genetic variation between the homoeologous A and D sub-genomes and thus genome sequencing of Upland cotton represent a unique challenge to generate high quality draft assembly.

## Sequencing Strategy

The genomic complexity, repetitive elements and polyploidy are bound to impede *de novo* sequencing and assembly using whole genome shotgun sequencing with NGS technologies alone. Generating massive amounts of sequence data rapidly does not address the difficulties of assembling complex plant genomes [1]. Use of physical map from BAC fingerprinting and genetic mapping data from molecular markers would help in associating the sequence data generated from BACs to individual chromosomes in Upland cotton.

To evaluate the significance of NGS technologies compared to traditional approaches and to derive an efficient sequencing strategy for polyploid genomes, we proposed a cost-effective BAC-pool sequencing approach (Figure 1). Selection of BAC clones in MTP allows combining the DNA from individual BACs in a contig into small pools (3-4 BACs/pool) without the necessity of barcoding during library preparation and to generate larger scaffolds during assembly process. With the first generation 454 GS FLX instrument (not Titanium chemistry), five such BAC-pools were sequenced to provide >30-fold coverage in both contigs. Additional Sanger sequencing data had helped to improve the scaffold length through the hybrid assembly process (Table 1).

### Sequence assembly and evaluation

Sequence assemblies were initially evaluated using 454 and Sanger datasets individually and also by combining the datasets for each BAC-pool. Despite high coverage (~35X) across all BAC-pools, the 454-pool assemblies mostly contained sequence contigs < 1Kb size (284 for Ctg-3301 and 448 for Ctg-465), while fewer (47 for Ctg-3301 and 53 for Ctg-465) sequence contigs were > 1Kb. Short read lengths produced by first generation Roche/454 GS20 FLX instrument deters generation of fewer and longer contigs and thus resulting in more gaps in the 454-assemblies. The assembled sequence contigs constituted 66% (~338.4Kb) and 75% (~674Kb) of the estimated sizes of Ctg-3301 (513.5Kb) and Ctg-465 (898.2Kb) respectively. Repetitive sequences in these contigs might have over-assembled to result in fewer contigs during assembly process. In this scenario, the assembly could have inserted few breaks and resulted shorter assembly sizes. By introducing the paired-end data in the Sanger assemblies, the number of gaps was relatively reduced.

We also evaluated a hybrid assembly approach to utilize the data from both 454 and Sanger datasets. Although a Sanger/pyrosequencing hybrid approach was evaluated earlier for *de novo* sequencing of marine microbial genomes [11], it did not include the read trimming and Phred-based assembly approach we now report. Since dissimilarities in the quality estimates and parameters for base calling between the two technologies create problems in assembly in 454-Sanger hybrid approach, selecting a minimum putative ‘Phred’ quality value of 16 to the 454 raw reads in a BAC-pool allowed to use them along with the Sanger reads from the respective BAC clones to generate Sanger-454 hybrid pool assemblies using ‘Phrap’. Our hybrid assembly process reduces the number of gaps and better complements the coverage in both sequencing methods to generate assemblies with fewer and longer sequence contigs. There was a 16% improvement over the 454 cumulative assembly of Ctg-3301 (~76.5% coverage), and 9% of Ctg-465 (~81.9% coverage), thus providing better coverage of the sequenced regions (File S1). The number of gaps in the hybrid assemblies was significantly reduced (~53%) while significantly increasing the length of the average contig size. Compared to initial 454 BAC-pool assemblies, the number of gaps were greatly reduced (>53%) in the BAC-pool hybrid assemblies and cumulative hybrid assembly sizes were also improved by 16.1% and 9.1% over 454 assemblies in Ctg-3301 and Ctg-465, respectively. In this study, hybrid assembly process performed little better in minimizing the number of gaps compared to Newbler.

**Figure 2:**
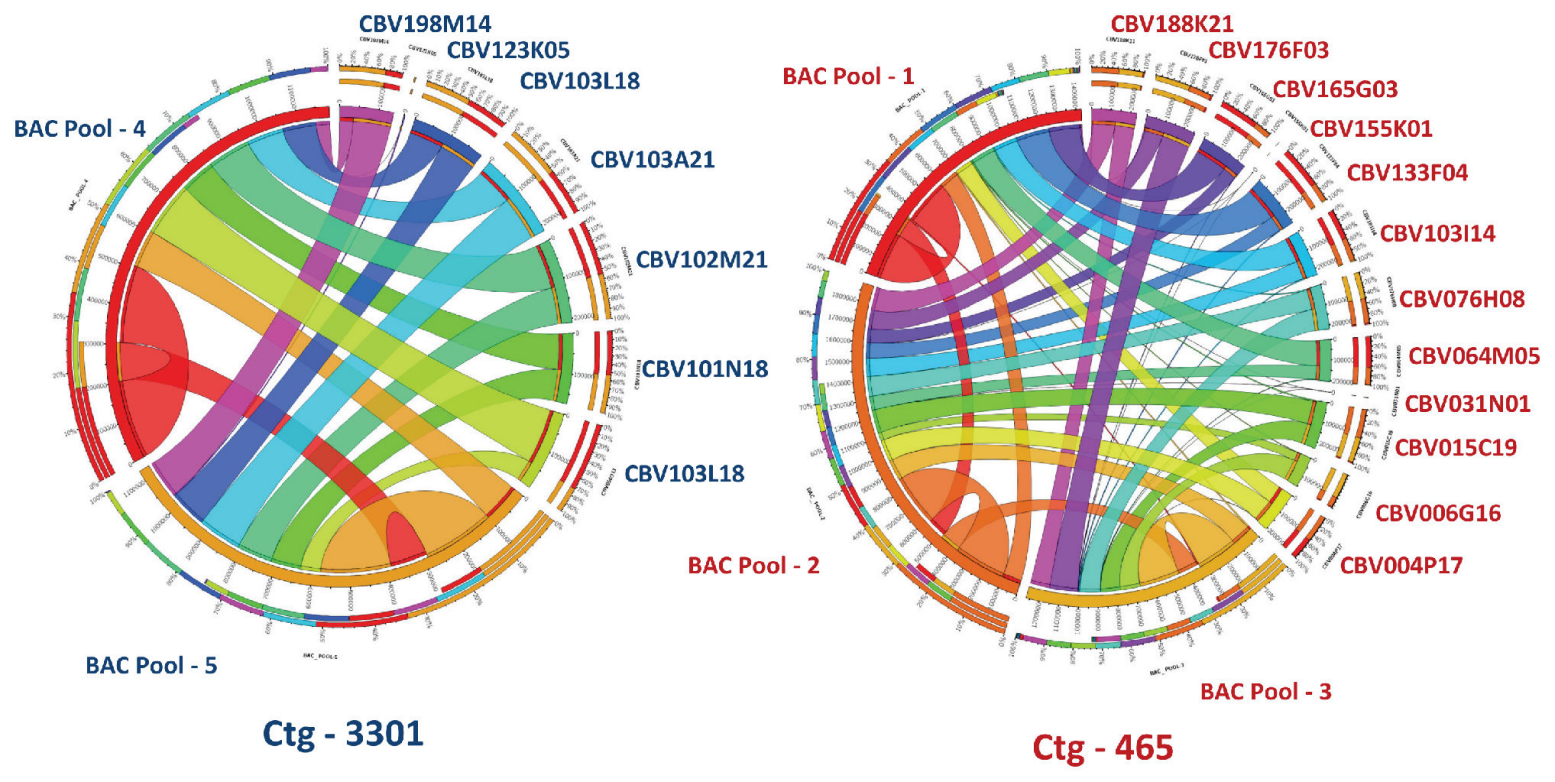
Diagrammatic representation of BAC-pools and their homology across the pools and respective BAC clone ABI assemblies in Ctg-3301 and Ctg-465.

Sequence assemblies were initially evaluated with BAC-end sequences (BES) to confirm the data from all BACs in the 454 and Hybrid pools along with individual BAC assemblies from Sanger reads. BES analysis revealed higher homology in hybrid assemblies compared to 454-pool and individual Sanger assemblies (File S2). Because the BAC-pools overlap with each other in each chromosomal segment, BES sequences were cross represented across the pools. For example, forward BES of CBV103I14 (Pool-1) was represented with 1002bp (Pool-2) match, while the reverse (r) BES of the same clone also was represented with 970bp match in Pool-2 and 990bp match in Pool-3. This explains that BAC clone CBV103I14 is extended up to Pool-3 in Ctg-465. Similar analogy with such cross-representation was used to predict the orientation of BAC clones under each contig region. Overlapping patterns of the BAC pools and with their respective ABI BAC assemblies in Ctg-3301 and Ctg-465were depicted in Figure 2. Interestingly, we also found significant sequence similarity of BESs from Ctg-3301 with the contigs of Ctg-465 and vice versa. This cross representation of BES between Ctg-3301 and Ctg-465 explained the degree of homoeology between chromosomes D12 and A12. For improved assembly reasons, the contigs from hybrid assembly were utilized for further data analyses.

### Repeat sequence analysis

GC content in both Ctg-3301 and Ctg-465 was ~35% suggesting that the Upland cotton genome is relatively AT-rich and it was in concordance with earlier studies [20]. Gene density in the sequences is usually affected by the repeat elements. Sequence analysis for repetitive elements revealed high proportion of LTR retrotransposon regions compared to other repeat elements. BAC-Pool 1 has relatively high repeat elements (16.5Kb) compared to Pool-2 (8Kb) and Pool-3 (10.3Kb) in Ctg-465 while BAC-pool 4 (11.7Kb) was repeat-rich than Pool-5 (6Kb). These repeat regions contained largely *Copia* and *Gypsy* retrotransposon elements in both Ctg-3301 and Ctg-465 further confirming their proliferation in polyploid cotton genome [37]. Significant homology was also observed in the repeat regions of Ctg-3301 and Ctg-465 revealing the conservancy of the repeat elements in both sub-genomes of tetraploid cotton (File S3).

### Sequence homology with cotton SSRs and unigenes

Comparison of Ctg-3301 and Ctg-465 with the existing sources (CN, EST, GSS) of *Gossypium* species at GenBank revealed highest homology with genome survey sequences in *G. hirsutum* and these significant hits were mostly in the repeat regions. Contigs in sequence assemblies were searched for SSR regions as they would help in designing new SSR markers for tagging the genes in these segments of A12 and D12 chromosomes. Pentanucleotide repeats were dominant across all pools (File S4). SSR marker information at CMD was used to identify the putative targets for the markers by *in silico* PCR and it disclosed 6 markers for Ctg-3301 and 5 markers for Ctg-465 (File S5). The marker fragment lengths of these markers were not mapped earlier to the A12 and D12 chromosomes.

A global assembly of cotton ESTs was earlier conducted by Udall et al. [38] and was used to annotate the unigene information in cotton. Xu et al [9] had developed integrated physical maps for chromosomes A12 and D12 and also were able to anchor 401 fiber unigenes and 214 non-fiber unigenes to chromosome A12; and 207 fiber unigenes and 183 non-fiber unigenes to chromosome D12 in cotton by overgo hybridization. Comparison of these anchored unigene/marker information would identify their physical location in the sequence of Ctg-3301 and Ctg-465. From the physical map of A12 chromosome, 21 unigene/markers were localized to Ctg-3301. Similarly, 34 unigenes/markers from D12 physical map were localized to Ctg-465. This information conforms with Xu et al [9] overgo study and also helps in directly anchoring these unigene sequences to these contigs for gene tagging.

### Gene identification and annotation

Deriving the protein coding information from Ctg-3301 and Ctg-465 facilitates the identification of the genes, their structure and functional significance. Using FGENESH, an *ab initio* protein prediction program, 137 protein coding regions were detected in Pool-1, 2, 3 of Ctg-465 and 77 protein coding regions were detected in Pool-4 and 5 of Ctg-3301. However, there were 71 and 109 non-redundant protein coding regions in Ctg-3301 and Ctg-465, respectively. Based on this information, the average gene space was 5.5Kb in Ctg-3301 and ~6.75Kb in Ctg-465. The annotated information on functional genes was obtained using BLAST2GO tools. A total of 48 proteins in Ctg-3301 and 48 proteins in Ctg-465 were annotated with functional gene information as well as GO IDs (File S6). Some of the genes (File S6) are involved in cellular metabolic processes, defense mechanisms, transcript regulation, transportation and other physiological processes. Further investigation of these genes would help in characterization of metabolic processes they were involved and their regulation.

In both Ctg-3301 and Ctg-465, several retroelement proteins such as retrotransposon *Ty1-copia* subclass, retrotransposon gag protein, retrotransposon *Ty3-gypsy* subclass, RNA-directed DNA polymerase (reverse transcriptase) polynucleotidyl ribonuclease h fold, pif-like transposase, gag poly protein were also identified through this analysis. This information provides an opportunity to study different classes of retroelements, their structure, regulation in addition to reveal the variation in these genes between A and D sub-genomes and role in genome evolution of Upland cotton. Several uncharacterized or hypothetical proteins detected in these contigs may help in investigating their function and regulation. A detailed summary of the genes identified in each BAC-pool, their exons, protein sequence and annotation was summarized in File S6.

### Comparison with *Arabidopsis* genome

**Figure 3:**
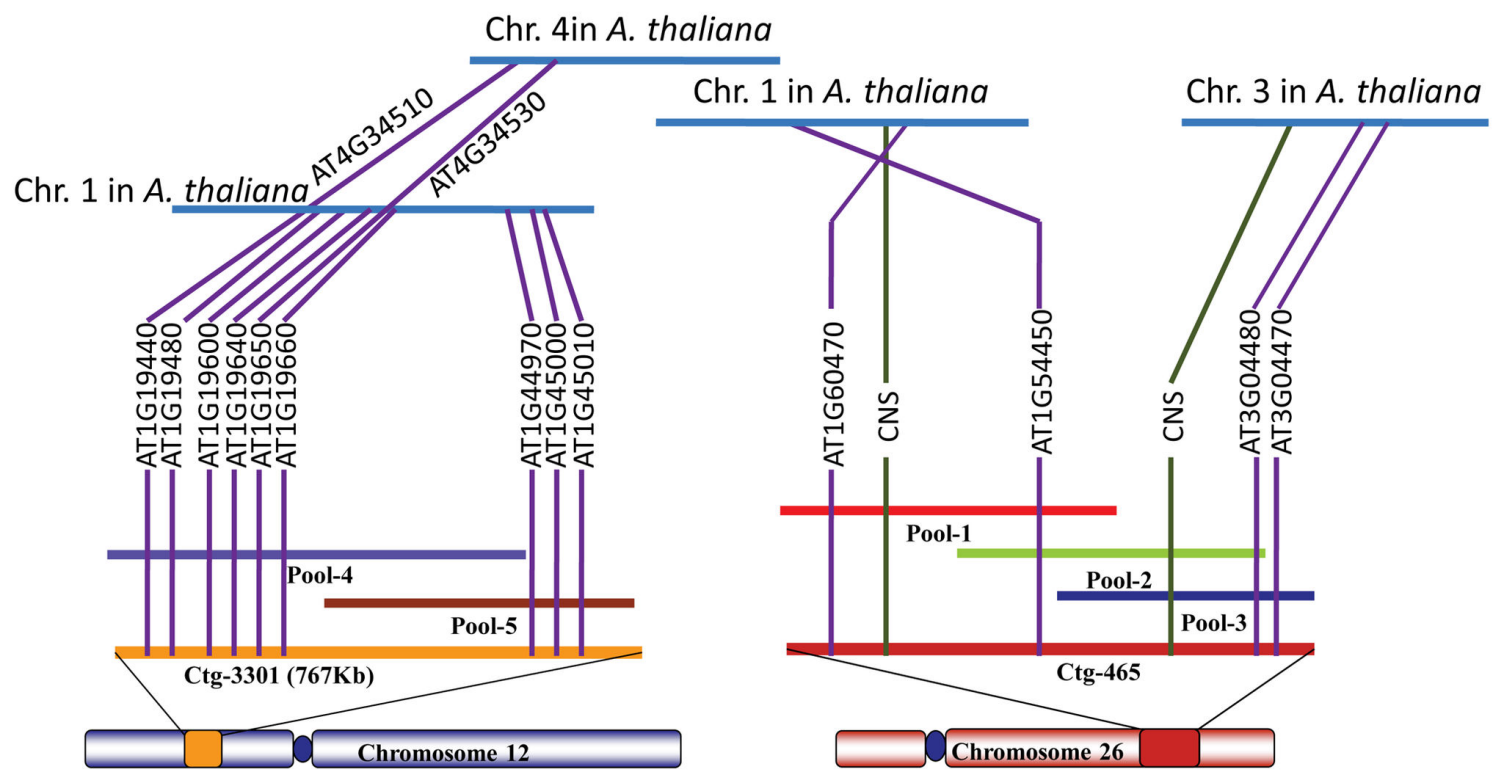
Comparative genomic analysis of Ctg-3301 (Chromosome A12) and Ctg-465 (Chromosome D12) of Upland cotton with *Arabidopsis* genome.

Previous studies indicated a high degree of homology between cotton and *Arabidopsis* genome, especially in gene order [33]. Comparing the sequences of Ctg-3301 and Ctg-465 with *Arabidopsis* genome helps in identifying the syntenic regions and also provides prospects to study the gene divergence through evolution. Genome VISTA tools [28] were used for genomic comparison of Ctg-3301 and Ctg-465 with *Arabidopsis* genome and a hypothetical diagrammatic representation of the analysis was presented in Figure 3.

The genes identified earlier in annotation process had significant homology with the functional genes in *Arabidopsis* in addition to having synteny. Ctg-3301 had high homology with chromosome 1, followed by chromosome 4 in *Arabidopsis*. We observed large syntenic regions along with microcollinearity in the gene order of Ctg-3301 with *Arabidopsis* chromosome 1 (cumulative length of ~85.9Kb) and chromosome 4 (cumulative length of ~25.4Kb). Few of the genes in Ctg-3301 such as 3-ketoacyl-CoA synthase (Chr1: AT1G19440 and Chr4: AT4G34510) and SEC 14 protein (Chr1: AT1G19650 and Chr4: AT4G34580) had significant homology with genes that were duplicated in both chromosome 1 and chromosome 4 of *Arabidopsis*, thus providing the additional evidence for the gene duplication events studied earlier [39].

Similarly, Ctg-465 had homology with chromosome 1 (cumulative length of ~15.9Kb) and chromosome **3** (cumulative length of ~35.6Kb) of *Arabidopsis*. Minor synteny was observed in functional genes of *Arabidopsis* chromosome **3**. We also observed high similarity with non-coding conserved nucleotide sequence (CNS) regions in both chromosome 1 and 3 of *Arabidopsis*. Such comparative analyses would aid in understanding the complex evolutionary processes in distant plant species from a common ancestor by gene conservation and gene termination. Details of the synteny and homology of both contigs with *Arabidopsis* genome was represented in File S7.

### Comparison with *G. raimondii* genome

Availability of draft genome of *G. raimondii*, the D-genome progenitor of allotetraploid cotton provided insight about cotton genome architecture [35,36] and gene content. Using MUMMER tools [40] Ctg-3301 and Ctg-465 were compared with gold standard draft genome sequence of *G. raimondii* [36] to detect syntenic regions (File S7). Significant synteny with scaffold-9 was detected from Ctg-3301(A-sub genome) while Ctg-465 (D-sub genome) had significant synteny with scaffold-13. Though both Ctgs are from homoeologous chromosomes A12 and D12, lack of syntenic association with the same scaffold region suggest duplication or translocation events of the Ctgs during the allotetraploid evolution. A large number of other significant random hits were detected specifically in scaffold 8-13 and these hit regions mostly included repeat regions.

## Conclusion

Whole genome sequence of Upland cotton would have higher impact and direct application for improving the cotton productivity compared to that of diploid progenitors. Despite the high complexity and repetitive nature of Upland cotton genome, shotgun sequencing approaches would create numerous problems in assigning the assemblies to highly homeologous chromosomes and thus deter the advantages of NGS platforms. To overcome the challenges of generating high quality genome sequence data in Upland cotton, we proposed a novel MTP-based BAC-pool sequencing method which assists in accurate association of sequence data to highly homoeologous chromosomes to generate a high quality draft tetraploid cotton genome sequence. We generated ~393Kb and ~735Kb data by an improved hybrid assembly process of 454 and Sanger reads from Ctg-3301 and Ctg-465 in A12 and D12 homoeologous chromosomes of Upland cotton. This is one of few studies reporting the sequencing of large chromosomal segments of Upland cotton along with a multitude of sequence analyses including gene identification and comparative genomics with *Arabidopsis* and with the draft *G. raimondii* genome. A BIBAC physical map of Upland cotton had been recently constructed [41] and genome sequence of A-genome progenitor *G. arboreum* will be released soon [42]. As NGS technologies are rapidly evolving with novel methods such as single molecule sequencing, nanopore sequencing etc., there is tremendous scope to sequence large genomes more efficiently. However, while these next-next-generation technologies mature, combination of traditional BAC-based sequencing with NGS technologies and leveraging the diploid progenitor genome sequences would provide the direction for sequencing the whole genome sequence of Upland cotton rapidly and accurately in the near future.

## Supporting Information

File S1 This file contains sequence assembly statistics and a comparative diagram of the cumulative assembly length of Ctg-3301 and Ctg-465 in both 454 and hybrid (454 reads + Sanger reads) assemblies with the estimated contig size.
(XLSX)

File S2 Diagrammatic representation of overlapping patterns between the BAC-pools in Ctg-3301 and Ctg-465 using Circos.
(XLSX)

File S3 Repeat content information across BAC-pool assemblies.
(XLSX)

File S4 Species-specific homology and simple sequence repeat information across BAC-pool assemblies.
(XLSX)

File S5 Comparative summary of unigene and marker information from A12 and D12 physical map with Ctg-3301 and Ctg-465 sequence assembly respectively.
(XLSX)

File S6 Gene content, translated protein, and gene annotation information in the sequence assemblies.
(XLSX)

File S7 Comparative homology & syntenic region summary of BAC-pool sequence assemblies with *Arabidopsis* and *G. raimondii* genomes.
(XLSX)
